# No effect of whole-hand water flow stimulation on skill acquisition and retention during sensorimotor adaptation

**DOI:** 10.3389/fnhum.2024.1398164

**Published:** 2024-06-07

**Authors:** Dat Le Cong, Daisuke Sato, Koyuki Ikarashi, Genta Ochi, Tomomi Fujimoto, Koya Yamashiro

**Affiliations:** ^1^Major in Health and Welfare, Graduate School of Niigata University of Health and Welfare, Niigata, Japan; ^2^Institute for Human Movement and Medical Sciences, Niigata University of Health and Welfare, Niigata, Japan; ^3^Sports Physiology Laboratory, Department of Health and Sports, Niigata University of Health and Welfare, Niigata, Japan

**Keywords:** sensorimotor adaptation, skill acquisition, memory retention, TMS, neuromodulation, whole-hand water flow, whole-hand water immersion

## Abstract

**Introduction:**

Repetitive somatosensory stimulation (RSS) is a conventional approach to modulate the neural states of both the primary somatosensory cortex (S1) and the primary motor cortex (M1). However, the impact of RSS on skill acquisition and retention in sensorimotor adaptation remains debated. This study aimed to investigate whether whole-hand water flow (WF), a unique RSS-induced M1 disinhibition, influences sensorimotor adaptation by examining the hypothesis that whole-hand WF leads to M1 disinhibition; thereby, enhancing motor memory retention.

**Methods:**

Sixty-eight young healthy participants were randomly allocated to three groups based on the preconditioning received before motor learning: control, whole-hand water immersion (WI), and whole-hand WF. The experimental protocol for all the participants spanned two consecutive days. On the initial day (day 1), baseline transcranial magnetic stimulation (TMS) assessments (T0) were executed before any preconditioning. Subsequently, each group underwent their respective 30 min preconditioning protocol. To ascertain the influence of each preconditioning on the excitability of the M1, subsequent TMS assessments were conducted (T1). Following this, all participants engaged in the motor learning (ML) of a visuomotor tracking task, wherein they were instructed to align a cursor with a target trajectory by modulating the pinch force. Upon completion of the ML session, final TMS assessments (T2) were conducted. All participants were required to perform the same motor learning 24 h later on day 2.

**Results:**

The results revealed that whole-hand WF did not significantly influence skill acquisition during sensorimotor adaptation, although it did reduce intracortical inhibition. This phenomenon is consistent with the idea that S1, rather than M1, is involved in skill acquisition during the early stages of sensorimotor adaptation. Moreover, memory retention 24 h after skill acquisition did not differ significantly across the three groups, challenging our initial hypothesis that whole-hand WF enhances memory retention throughout sensorimotor adaptation. This could be due to the inability of whole-hand WF to alter sensorimotor connectivity and integration, as well as the nature of the plastic response elicited by the preconditioning.

**Discussion:**

In conclusion, these findings suggest that although whole-hand WF attenuates intracortical inhibition, it does not modulate skill acquisition or motor memory retention during sensorimotor adaptation.

## Introduction

We often adapt to changes in our daily lives. For instance, when we pick up an object of uncertain weight, step on a slippery surface, or navigate in difficult conditions, we quickly adjust our movement dynamics. This ability to adjust our movements quickly and flexibly is termed sensorimotor adaptation (Reuter et al., 2022). Sensorimotor adaptation generally unfolds in two distinct stages. During the first stage, errors prompt continuous refinement of motor commands, leading to incremental changes in movements. With practice and feedback over time, motor skills are enhanced and stabilized. Multiple brain regions, including the sensorimotor cortex, basal ganglia, and cerebellum, are involved in these acquisition, consolidation, and retention processes (Doyon and Benali, 2005). The somatosensory cortex (S1) plays an essential role in early adaptation, including skill acquisition and consolidation (Bernardi et al., 2015; Kumar et al., 2019). Subsequently, the plasticity of the primary motor cortex (M1) is known to be involved in the retention phase (Hadipour-Niktarash et al., 2007; Hamel et al., 2017). Therefore, understanding the influence of the neural states of the S1 and M1 on sensorimotor adaptation is a topic of great interest.

Repetitive somatosensory stimulation (RSS) is a technique that increases sensory input through sustained tactile stimulation (Veldman et al., 2014), commonly used to modulate the neural states of S1 and M1. This is substantiated by the existence of direct connections between S1 and M1, providing a neuroanatomical basis for the interdependence of sensory and motor functions (Jones, 1983; Krakauer et al., 2019). From a behavioral perspective, the close connection between somatosensory and motor functions is also evidenced by the motor consequences of anesthesia applied to cutaneous afferents and the effects of muscle afferent stimulation via vibration (Gentilucci et al., 1997; Rosenkranz and Rothwell, 2012; Pfenninger et al., 2024). Previous studies have shown that several RSS-induced neurophysiological changes, including expanded cortical representations of the stimulated body site (Golaszewski et al., 2002; Wu et al., 2005), induce long-term potentiation (LTP)-like plasticity indicated by increased corticospinal excitability (Ridding et al., 2001; Kaelin-Lang et al., 2002; Yildiz and Temucin, 2023) and intracortical disinhibition (Classen et al., 2000; Sato et al., 2014b). Such RSS-induced plasticity of the central nervous system is likely mediated by use-dependent, LTP-like mechanisms (Kaelin-Lang et al., 2002), which closely resemble the mechanisms proposed to underlie improvements in motor performance following motor skill training (Bütefisch et al., 2000; Cantarero et al., 2013). Moreover, studies have reported that RSS promotes improved skill acquisition and consolidation in healthy individuals (Veldman et al., 2018) and patients with stroke (Celnik et al., 2007). However, comparable results have shown no benefit of RSS in healthy individuals (Négyesi et al., 2018). Therefore, the influence of the RSS on sensorimotor adaptation, including skill acquisition and retention, remains controversial.

Several RSS modalities have been reported, including electrical and mechanical stimulation (Veldman et al., 2014, 2018). In this context, we have examined the effects of a safe and unique stimulation method using water immersion (WI) (Sato et al., 2013, 2014a,b). Currently, WI is part of the rehabilitation regimen for orthopedic, cardiovascular, and respiratory disorders. Partial whole-hand WI has been used as a therapeutic agent to alleviate edema, improve blood flow (Fothergill et al., 1998), and relieve pain (Kakigi, 1994). However, the scientific evidence for improving movement through sensorimotor adaptation is limited. Recently, we developed a novel RSS using water flow (WF) called “whole-hand WF.” (Sato et al., 2014b; Le Cong et al., 2022) Unlike general local electrical stimulation, this RSS can provide sustained stimulation over a large site on the hand (Godde et al., 1996; Rocchi et al., 2017). Besides the tactile and pressure sensory inputs from water, it has also been confirmed to induce skin vibration at approximately 70 Hz (Sato et al., 2014b). Previous neurophysiological studies have found that whole-hand WF induces neural disinhibition in M1 (Sato et al., 2014b) and not in S1 (Le Cong et al., 2022), whereas whole-hand WI (involving only the hand in water) and non-immersed controls had no effect in either M1 or S1. The discrepancy in the response to whole-hand WF between M1 and S1 is presumably due to the lower plasticity of S1 compared to M1 (Nakatani-Enomoto et al., 2012). Considering these distinct responses to whole-hand WF between S1 and M1 (Sato et al., 2014b; Le Cong et al., 2022) and that M1 involves memory retention during sensorimotor adaptation but not acquisition and consolidation (Hamel et al., 2017), whole-hand WF-induced plasticity in M1 may modulate motor retention during sensorimotor adaptation.

This study aimed to investigate whether whole-hand WF, inducing M1 disinhibition, alters sensorimotor adaptation. Previous studies have shown that the M1 is not involved in motor skill acquisition during sensorimotor adaptation (Baraduc et al., 2004; Richardson et al., 2006). However, numerous evidence exists that M1 modulates motor memory retention (Richardson et al., 2006; Hadipour-Niktarash et al., 2007; Hamel et al., 2017; Ohashi et al., 2019). Richardson et al. (2006) showed that preconditioning of repetitive transcranial magnetic stimulation (rTMS) to M1, which is long-term depression (LTD)-like plasticity, impairs motor memory retention and not acquisition during sensorimotor adaptation. Based on these results, we hypothesized that whole-hand WF induces M1 disinhibition, facilitating motor memory retention.

## Materials and methods

### Participants and group assignment

We used the General Linear Mixed Model Power and Sample Size (GLIMMPSE) 3.1.2 tool to estimate the required sample size for this study (Kreidler et al., 2013). We determined the sample needed to have 80% power to detect a medium-sized group × block interaction and set alpha to 0.05. GLIMMPSE calculated the required sample size of 54 (18 per group). Therefore, we recruited 68 right-handed young, healthy volunteers (33 male and 35 female) to participate in this study. Participants with a history of neurological or psychiatric disorders or the use of psychoactive medications were excluded. None of the participants had previously been exposed to training in visuomotor learning tasks. This study was approved by the Ethics Committee of Niigata University of Health and Welfare, Japan (approval number: 18958-221207). All the experiments conformed to the principles of the Declaration of Helsinki. All the participants provided written informed consent before starting the experiment. All participants were randomly divided into three groups to avoid sex and age bias: control (CON, *n* = 22), whole-hand WI (*n* = 23), and whole-hand WF (*n* = 23). However, the investigator decided to exclude eight participants from this study because they exhibited an insufficient response to TMS, failing to elicit a motor evoked potential (MEP) of 1 mV even at 85% of maximum stimulator output (MSO) (CON group: 2, WI group: 3, WF group: 3). The participants received different preconditioning depending on the group (described in detail below).

### Procedures

**Figure 1:**
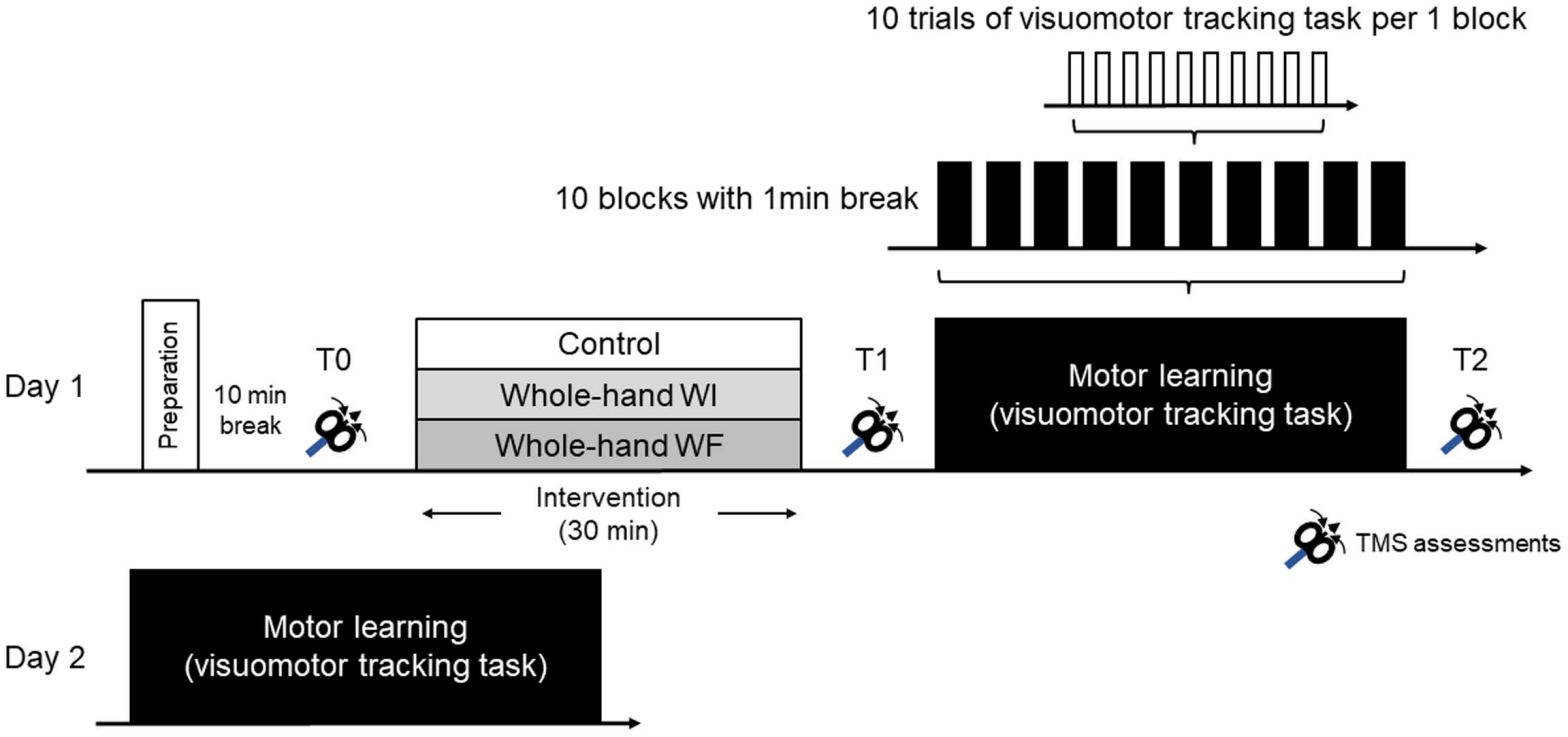
Experimental procedure in the present study.

Figure 1 illustrates the experimental procedure used in this study. The experiment for all the participants consisted of two sessions on consecutive days. On the first day (day 1), after preparation for TMS assessments and a 10 min break, baseline TMS assessments (T0) were conducted before each preconditioning. Subsequently, each group underwent different preconditioning protocols lasting 30 min. To assess the effect of each preconditioning on M1 excitability, TMS assessments were performed after each preconditioning (T1). Motor learning (ML) in the visuomotor tracking task was conducted, followed by TMS measurements (T2). All participants repeated the same ML task 24 h later on day 2.

### Preconditioning

Each preconditioning session followed the same methodology outlined in our previous study (Sato et al., 2014b). Participants were instructed to place their right hand in a sluicing device (Japan Aqua Tec, Sasebo, Nagasaki, Japan) and maintain relaxation throughout the preconditioning period. The left hand was placed on a soft support beside the body and was kept relaxed. The hands were fixed in the same position for all preconditioning sessions using a belt to prevent muscle contractions. The participants were instructed to fixate their gaze on the wall facing them throughout the experiment to divert their attention from their right hand. Preconditioning sessions, each lasting 30 min, consisted of non-immersion for the CON group, whole-hand WI for the WI group, and whole-hand WF for the WF group. The duration of preconditioning was determined based on the following previous results: (1) at least 15 min was required to attenuate short-interval intracortical inhibition (SICI) by whole-hand WF (Sato et al., 2014b) and (2) inconsistent results were obtained about the effects of RSS on sensorimotor adaptation with durations set at 20–25 min (Celnik et al., 2007; Veldman et al., 2016). During whole-hand WI and WF, water reached up to the right forearm, and whole-hand WF was directed at the palm of the right hand using a sluicing device at 40 L/min. Participants in the CON group simply placed their hands on the device for 30 min without water. For each preconditioning session, the ambient temperature was 29°C ± 1°C, and the water temperature was 33°C ± 1°C. The ambient and water temperatures were modulated to avoid changes in the skin temperature.

### TMS assessments and electromyographic recordings

TMS was used to assess preconditioning and ML-related M1 plasticity. To evaluate corticospinal excitability, inhibitory and excitatory circuits, sensorimotor integration, MEPs, SICI and short-interval intracortical facilitation (SICF), and short-latency afferent inhibition (SAI) were measured at rest before each preconditioning (T0) and before and after ML (T1 and T2).

Surface electromyographic (EMG) recordings were acquired from the right first dorsal interosseous (FDI) muscle and abductor pollicis brevis (APB) muscles of the right hand with 9 mm diameter silver-silver chloride (Ag-AgCl) surface electrodes. Only the EMG data from the FDI muscle were used to analyze M1 plasticity because it is the primary operating muscle for the present motor task. The active electrode was placed over the muscle belly, and the reference electrode was placed over the interphalangeal joint of the index finger and thumb. The raw signal was amplified, band-pass filtered between 5 Hz and 2000 Hz (AB-611JMG, Miyuki Giken, Tokyo, JPN), and transferred through a Multi Stim Tracer System (Multi Stim Tracer System, Medical Try System, Tokyo, JPN) to a personal computer for offline analysis. All electrodes were covered with a transparent film (Tegaderm Hydrocolloid Dressing, 3 M Japan, Tokyo, JPN) for waterproofing. Waterproofing was applied before the experiment and removed after the final assessment in all experiments.

TMS was conducted using a figure-of-eight-shaped coil with an internal wing diameter of 7 cm, connected to two Magstim 200^2^ stimulators (Magstim, Dyfed, United Kingdom). Each stimulation was performed with the BiStim configuration in this study. The coil’s handle pointed backward and 45° laterally to the interhemispheric line, inducing an anteriorly directed monophasic current in the brain, and was optimally positioned to obtain MEPs in the FDI muscle. The coil’s target position relative to the brain anatomy was confirmed using a frameless TMS navigation system (Brainsight, Rogue Resolution, United Kingdom). Since individual MRI data could not be obtained, we identified the stimulus location where MEPs were stably elicited by low-intensity TMS and recorded its positional coordinates in a template brain. Based on these data, the stimulus location identified was stimulated with a displacement of less than 2 mm each time throughout all experiments.

In the preparation, resting and active motor thresholds (RMT and AMT, respectively) were measured to determine the stimulus intensity of the conditioned stimuli (CS) for SICI and SICF. RMT was the lowest TMS intensity required to elicit an MEP with a peak-to-peak amplitude greater than 50 μV in 5 out of 10 consecutive stimulations when the participants were at rest (Rossi et al., 2009). The AMT was defined as the lowest intensity required to evoke an MEP of 200 μV in more than five of 10 consecutive trials when the participants maintained approximately 10% contraction of the target muscle (Ridding et al., 1995). The participants maintained 10% maximal voluntary contraction (MVC) by checking the EMG output on the monitor; weak contractions during the AMT measurement were performed in the neutral position with the force transducer used for the motor task. Additionally, the somatosensory evoked potential (SEP) was measured to determine the inter-stimulus interval (ISI) when measuring SAI. SEP was recorded using active electrodes placed at C3’ (located 2 cm posterior to C3). A reference electrode was placed at Fz, with the ground electrode at AFz. SEP was measured with an intensity that showed a visible muscular twitch in the thumb. The latency of the N20 peak was detected from the obtained SEP waveform in each participant.

Corticospinal excitability was assessed using the MEP amplitude produced by single-pulse TMS. TMS intensity was set to elicit an unconditioned MEP in the relaxed right FDI with approximately 1 mV (0.9–1.1 mV) peak-to-peak amplitude at T0 assessment. Intracortical inhibitory and excitatory circuits were assessed using SICI and SICF, which are involved in the indirect wave 3 (I3 wave) (Rusu et al., 2014). Paired-pulse TMS was administered through the same TMS coil over the left motor cortex, and the effect of CS on the test stimulus (TS) was measured. To measure SICI, the CS intensity was set at 90% of the AMT and applied before TS with a 3 ms ISI (Kujirai et al., 1993). For SICF, the CS intensity was set at 90% of the RMT and applied 3 ms after the TS (Ziemann et al., 1998). Additionally, sensorimotor integration related to the I3 wave through cholinergic (Ch) and GABAergic neural activities was assessed by measuring the SAI (Tokimura et al., 2000; Turco et al., 2021). ISI between the CS and TS for the SAI was set to a latency of N20 plus 2 ms. As mentioned previously, the N20 latency was determined for each participant based on the SEP data. The CS was the median nerve electrical stimulation at 2.5 times the ST intensity. The two electrodes were attached with double-faced tape and covered with a waterproof transparent film (Tegaderm Hydrocolloid Dressing; 3 M Japan, Tokyo, Japan). Corticospinal excitability at each measurement point (T1 and T2) was evaluated by averaging the MEP amplitudes obtained from 20 single-pulse TMS at the intensity eliciting MEPs of 0.9–1.1 mV at T0. Based on these data, TS intensity at each measurement point was determined by confirming that the amplitude of MEPs obtained at that time was 0.9–1.1. TS intensity was set to elicit an unconditioned MEP in the relaxed right FDI with approximately 1 mV (0.9–1.1 mV) peak-to-peak amplitude in all stimulus paradigms. The assessment block comprised 60 trials, including 15 for each stimulus paradigm (SICI, SICF, SAI, and TS alone). Fifteen pulses were delivered to each stimulus paradigm with an inter-trial interval (ITI) of 4–5 s, and the stimulus paradigms were randomly administered. SICI, SICF, and SAI were calculated as the ratios of the conditioned MEP amplitude to the unconditioned MEP amplitude.

### Motor learning for sensorimotor adaptation

**Figure 2:**
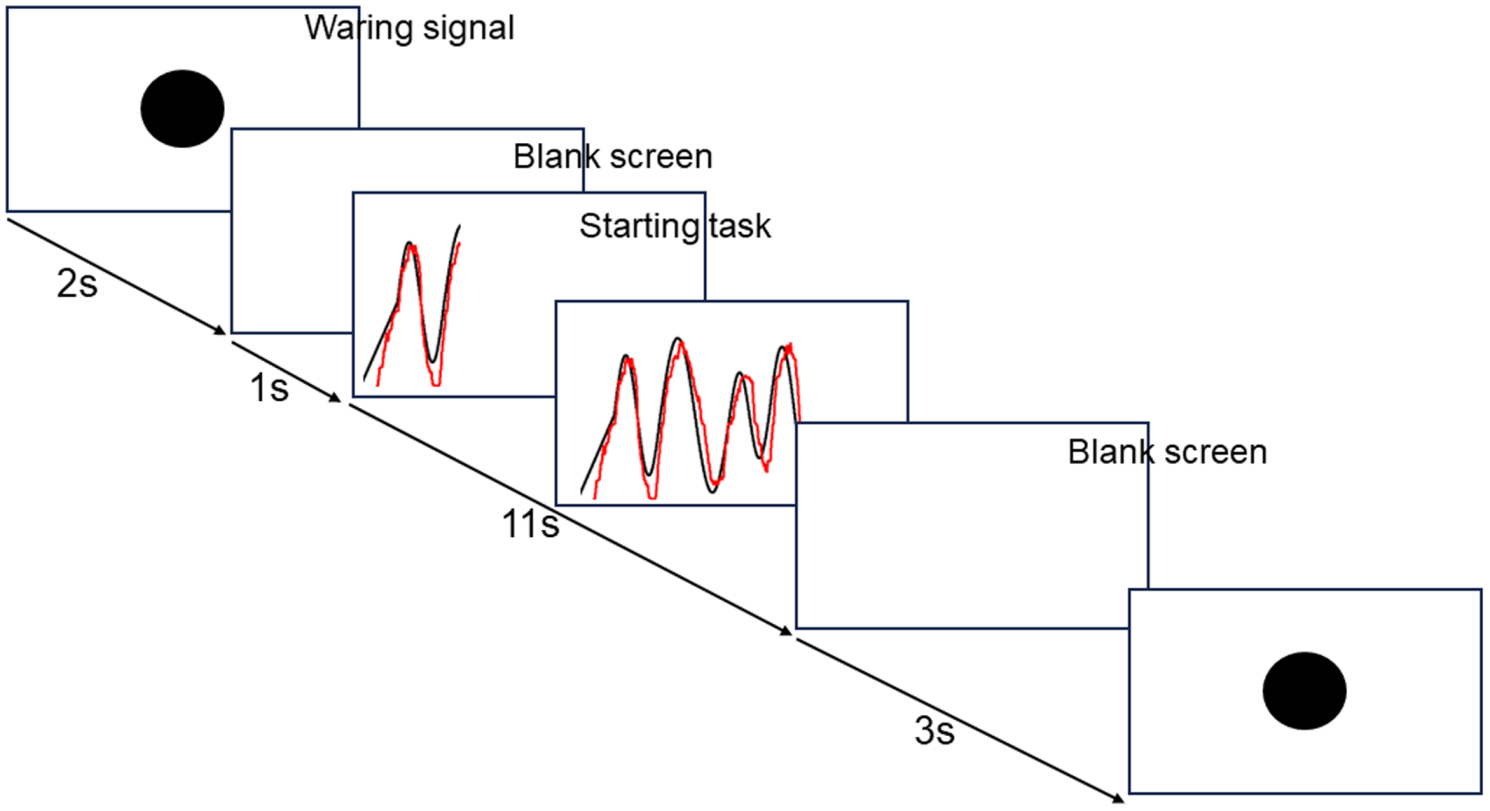
The flow of visuomotor tracking task.

The ML session consisted of 100 trials, 10 trials × 10 blocks with a 1 min break between blocks. The visuomotor tracking task was performed using a custom-built computer program (DASYLab version 2016; Measurement Computing Corp.) for ML (Figure 2). Each trial lasted 17 s. In each trial, a warning signal and a blank screen were presented for 2 s and 1 s, respectively. Subsequently, a black target line appeared from the bottom left corner of the monitor and moved to the right side for 11 s. The target line, a sinusoidal curve ranging from 5 to 15% of the subject’s maximum force measured before ML, varied in amplitude with each cycle; thereby, altering its velocity. Simultaneously, a red line appeared at the same point as the black line and moved to the right side of the monitor for 11 s. The first 1 s was excluded from the task performance evaluation. A blank screen was presented for 3 s until the next trial commenced. The participants were instructed to adjust the red line to the target black line on the screen by controlling the force transducer in their hands. By pinching the force transducer, the participants moved the red line vertically on the screen in real-time and proportionally to the applied force. Increasing the pinch force produced an upward red line movement, whereas decreasing the pinch force produced a downward red line movement. Although the flexor pollicis brevis is the primary muscle for pinching, the FDI and APB are also involved in a synergistic way; therefore, pinching was used in this study. Participants were also instructed to relax when not performing the motor task. The present visuomotor task required a longer execution time than previous studies, reflecting the complexity of perception-action processes involved in daily life and sports training.

Each block consisted of the same 10 trials throughout. Task performance was assessed by calculating the mean area of deviation from the target black line across 10 trials per block, ensuring no intergroup differences in baseline data. Task performance in each block was evaluated as a ratio to performance in the first block on day 1; task performance on day 1 was classified as acquisition, and performance on day 2 was categorized as retention.

### Statistical analysis

PASW statistics software version 27 (SPSS; IBM, Armonk, NY) was used for the present analysis. Unless otherwise stated, data are displayed as mean ± standard error of the mean (SEM). Normality was assessed using the Shapiro–Wilk test. Statistical significance was set at *p* < 0.05.

A two-factor linear mixed model analysis with repeated measures (LMM_RM_) using the MIXED command was used to probe whether skill acquisition (on day 1) and retention (on day 2) during sensorimotor adaptation differed depending on the three preconditioning: CON, WI, and WF. The LMM_RM_ was used to compare the normalized task performance between blocks as an intra-subject factor and between groups as an inter-subject factor.

LMM_RM_ was employed to examine the impact of ML-induced M1 plasticity on corticospinal excitability, as well as inhibitory and excitatory intracortical excitability in M1 before and after ML, under the influence of the three preconditioning methods: CON, WI, and WF. The LMM_RM_ compared the unconditioned MEP amplitude induced by single-pulse TMS, SICI, SICF, and SAI across time points (T0, T1, and T2) as intra-subject factors, with groups as inter-subject factors. All random subject effects (intercepts and slopes) were included, ensuring model convergence (Barr et al., 2013). Model fit was optimized by testing different covariance structures, with the structure providing the best fit (assessed using the Bayesian Schwartz Criterion; BIC) within a convergent final model. To determine the optimal model, BIC was compared, and the model with the lowest BIC was chosen. All main effects and interactions were performed using custom contrasts with Bonferroni corrections. The number of Bonferroni corrections was determined by the number of repeated measurements: 10 for motor tasks and three for neurophysiological measurements.

## Results

### Skill acquisition and retention during sensorimotor adaptation

**Figure 3:**
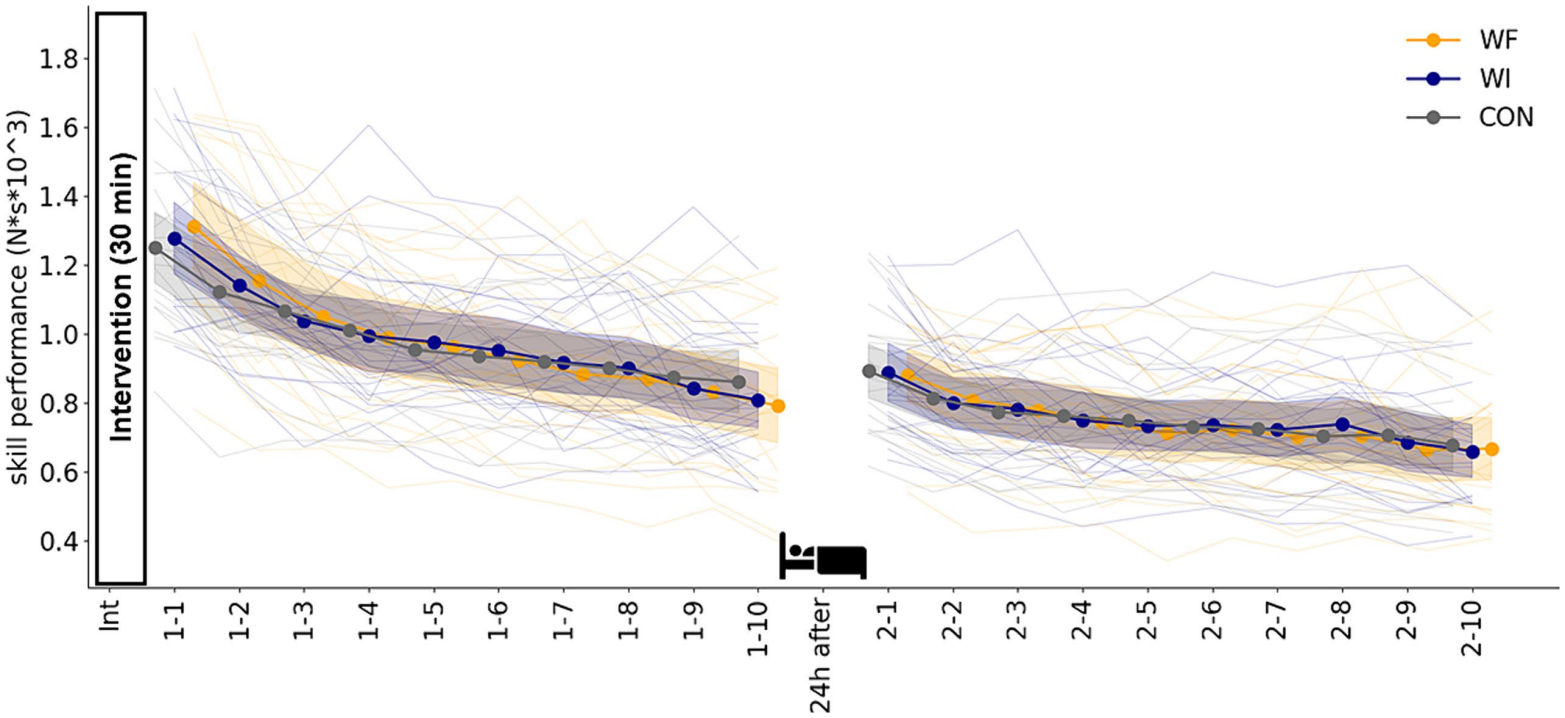
Skill performance during sensorimotor adaptation in each group. No significant difference was observed in skill acquisition on day 1 or retention on day 2 among the three groups. Filled circles and shaded areas represented the mean and 95% confidence interval (CI) for each group. Thin lines showed individual data for all participants. Yellow, dark blue, and grey presented WF, WI, and CON groups, respectively. WF, water flow; WI, water immersion; CON, control.

Figure 3 shows the skill acquisition (day 1) and retention (day 2) during sensorimotor adaptation in each group. The LMM_RM_ revealed a significant main effect of blocks on day 1 (*F*[9,171.349] = 136.165, *p* < 0.001) and on day 2 (*F*[9,214.854] = 45.954, *p* < 0.001). No significant interaction was observed between the group and block (day 1: *F*[18,171.349] = 1.016, *p* = 0.445, day 2: *F*[18,214.854] = 0.449, *p* = 0.975) and the main effect of the group (day 1: *F*[2,50.343] = 1.943, *p* = 0.154, day 2: *F*[2,81.573] = 1.112, *p* = 0.334).

### Neurophysiological data

**Figure 4:**
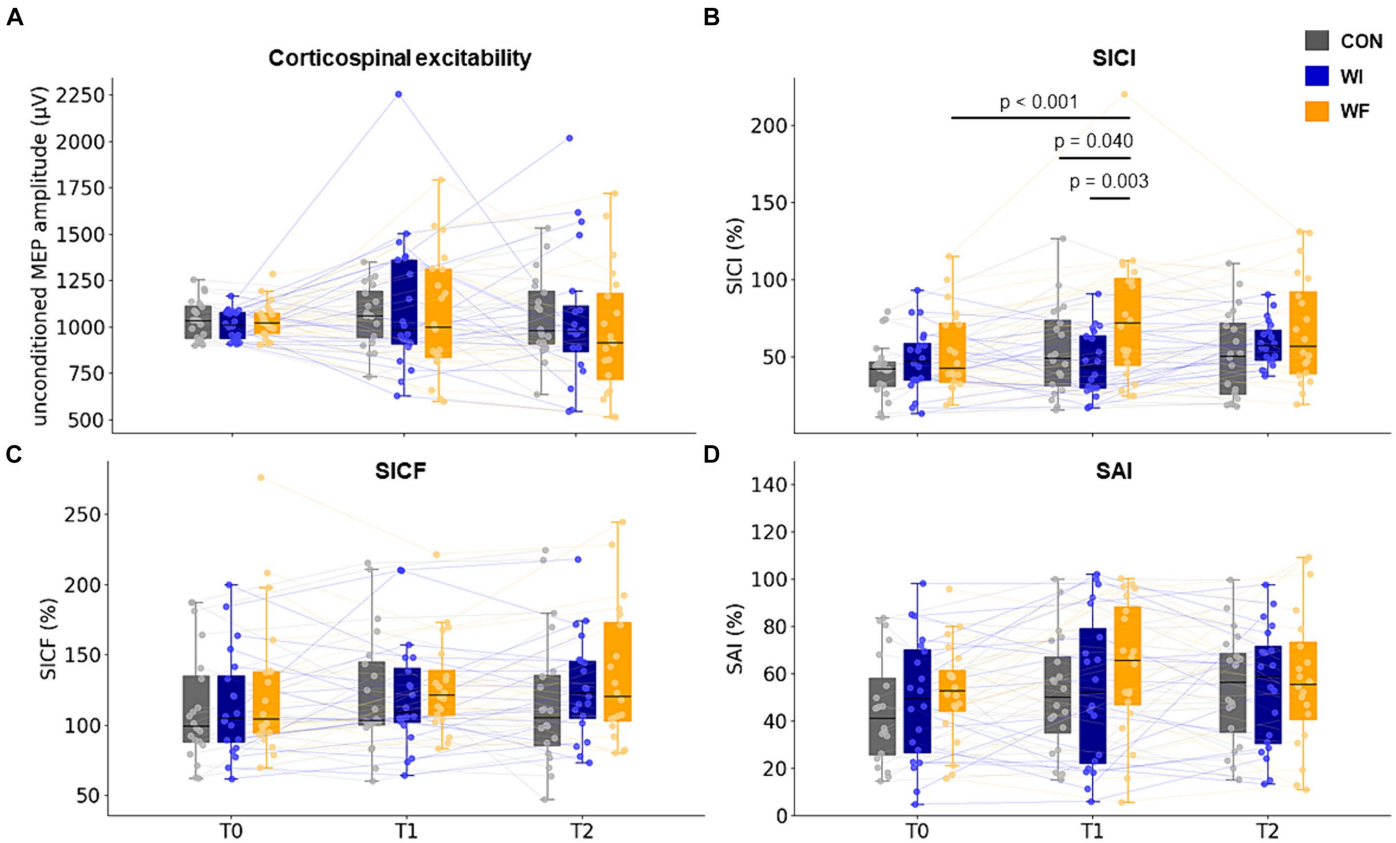
Change in corticospinal and intracortical excitability in M1. SICI significantly decreased after whole-hand WF but not after other preconditioning. No significant differences were observed between the groups throughout the experiment (T0 to T2). T0, T1, and T2 represented the time points of the TMS assessments: baseline, immediately after each preconditioning, and after motor learning, respectively. The filled circles and thin lines showed individual data for all participants. Yellow, dark blue, and grey presented WF, WI, and CON groups, respectively. WF, water flow; WI, water immersion; CON, control; M1, primary motor cortex. SICI, short-interval intracortical inhibition; SICF, short-interval intracortical facilitation; SAI, short latency afferent inhibition.

Table 1 shows the results of neurophysiological assessments at T0. No significant differences were observed in any of the indicators. Figure 4 shows the results of the TMS assessments in each group. LMM_RM_ found significant interaction between time points (T0, T1, and T2) and groups (prior preconditioning) for SICI (*F*[4,66.754] = 3.355, *p* = 0.015). However, no significant interaction was observed for unconditioned MEP amplitude (*F*[4,93.791] = 0.415, *p* = 0.798), SICF (*F*[4,102.422] = 0.540, *p* = 0.707), and SAI (*F*[4,110.833] = 0.390, *p* = 0.816). Additionally, no significant main effect of group was found for any of the TMS assessments (unconditioned MEP amplitude: *F*[2,64.474] = 0.188, *p* = 0.829; SICI: *F*[2,53.609] = 2.988, *p* = 0.059; SICF: *F*[2,79.931] = 0.724, *p* = 0.488; SAI: *F*[2,64.091] = 0.626, *p* = 0.538). A significant main effect of time point was observed in SICI (*F*[2,66.754] = 6.966, *p* = 0.002) and SAI (*F*[2,110.832] = 3.664, *p* = 0.029), with no significant effect seen in unconditioned MEP (*F*[2,93.474] = 1.569, *p* = 0.214) amplitude and SICF (*F*[2,102.422] = 2.803, *p* = 0.065). Post-hoc comparisons revealed significant SICI disinhibition after whole-hand WF (*p* < 0.001). Moreover, significant differences were observed in the SICI at T1 in the WF group relative to the WI and CON groups (WI, *p* = 0.003; CON, *p* = 0.040).

**Table 1:** Neurophysiological assessments at T0.

		CON group			WI group			WF group		
ST	(mA)	2.63	±	0.26	2.07	±	0.25	3.00	±	0.41
MT	(mA)	10.54	±	0.55	9.05	±	0.66	10.61	±	0.71
N20 latency	(ms)	18.25	±	0.32	18.58	±	0.17	18.75	±	0.23
RMT	(%MSO)	56.10	±	1.47	55.55	±	1.59	54.40	±	1.77
AMT	(%MSO)	49.40	±	1.48	49.90	±	1.51	49.55	±	1.82
TS_1mV	(%MSO)	65.10	±	2.01	64.90	±	1.90	63.80	±	2.31

CON, control; WI, water immersion; WF, water flow; ST, sensory threshold; MT, motor threshold; RMT, resting motor threshold; AMT, active motor threshold; TS, test stimulus.

## Discussion

This study investigated whether whole-hand WF, inducing M1 disinhibition, alters sensorimotor adaptation by testing the hypothesis that whole-hand WF induces M1 disinhibition, which facilitates motor memory retention. The main findings of this study indicate that whole-hand WF did not modulate skill acquisition or facilitate motor memory retention during sensorimotor adaptation despite a decrease in intracortical inhibition assessed by SICI.

The results showed similar skill acquisition in all groups, indicating that prior whole-hand WI and WF did not affect skill acquisition. This could be explained by the somatosensory experience accompanying the increased excitability in S1, which is necessary for skill acquisition during the early stages of ML (Darainy et al., 2013; Bernardi et al., 2015; Kumar et al., 2019). A previous study examining excitability in the S1 and M1 during sensorimotor adaptation found that skill acquisition initially induces an increase in S1 excitability, followed by an increase in M1 excitability (Ohashi et al., 2019). These results are consistent with the idea that plasticity in S1 is an integral component of the early stages of motor skill learning. Several studies have reported that the prior facilitation of S1 excitability facilitates skill acquisition during sensorimotor adaptation (Celnik et al., 2007; Veldman et al., 2018). In contrast, our previous study found that whole-hand WI and WF did not affect S1 excitability (Le Cong et al., 2022), although we did not directly measure S1 excitability in this study. Based on previous results, whole-hand WI and WF did not influence skill acquisition during sensorimotor adaptation because they did not affect S1 excitability.

Although previous studies have reported that a disrupted M1 does not influence skill acquisition (Richardson et al., 2006; Darainy et al., 2023), the repeated measures correlation analysis performed in this study showed that intracortical disinhibition assessed by SICI and SAI was significantly related to improved skill acquisition. This analysis could provide novel insights into the relationship between skill acquisition and prior neural activity in M1. Both previous and present studies have shown that M1 plasticity induction by preconditioning does not contribute to skill acquisition during sensorimotor adaptation from the comparison of changes in skill performance between each preconditioning. However, skill acquisition involves extensive neural networks (Gedankien et al., 2017) and complex intracortical and intercortical signaling (Paparella et al., 2020), potentially complicating the effects of preconditioning. Considering the robust relationship between the direction of the prior whole-hand WF-induced M1 plastic response and skill acquisition in this study, in addition to examining the relationship between physiological parameters at a temporal point and the amount of change in those parameters and behavioral data, examining the direction of change in each parameter is crucial.

Consistent with the process of skill acquisition, memory retention 24 h after skill acquisition did not exhibit any variance among the three groups, contradicting our initial assumption that whole-hand vibration facilitation (WF) can augment memory retention during sensorimotor adaptation phases. Although neural plasticity induced by sensorimotor adaptation is widely distributed, the consistency that M1 has a crucial role in motor memory retention has been obtained. This relationship between M1 excitability and memory retention was evident in experiments that modulated M1 excitability preceding (Richardson et al., 2006) and after (Muellbacher et al., 2002) acquisition, as well as during the consolidation phase (Hadipour-Niktarash et al., 2007; Hamel et al., 2017). Specifically, Richardson et al. (2006) employed rTMS to disrupt the M1 before skill acquisition, revealing that rTMS resulted in diminished performance upon reevaluation 24 h later; thereby, suggesting a critical role of the M1 in motor memory retention. Consequently, we postulated that whole-hand WF before skill acquisition could enhance motor memory retention 24 h later, as this preconditioning has been reported to attenuate intracortical inhibition in the M1 (Sato et al., 2014b). Whole-hand WF induces proprioceptive input from muscle spindles and cutaneous input from water flow, unlike whole-hand WI, which induces cutaneous input due to hydrostatic pressure alone (Sato et al., 2014b). This proprioceptive input, like vibration, has a greater effect on the cortical circuit controlling SICI than cutaneous input (Rosenkranz and Rothwell, 2012). Therefore, the attenuation of SICI was observed solely in the whole-hand WF group, consistent with previous research. Nevertheless, our findings did not demonstrate superior motor memory retention in the WF group relative to the other groups. A plausible explanation for these findings, which contradict our hypothesis, is the inability of whole-hand WF to modify sensorimotor connectivity and integration. Previous research has indicated that RSS can bolster motor memory retention, suggesting that enhanced sensorimotor neural connectivity correlates with improved memory retention (Négyesi et al., 2018). From these results, the authors claimed that RSS-induced facilitated retention may arise from the reinforcement of synaptic connectivity within the sensorimotor cortex, aligning with data showing gradual enhancements in motor network connectivity following skill acquisition (Della-Maggiore et al., 2017). Furthermore, an increase in the SAI, which assesses sensorimotor integration, has been associated with greater motor memory retention (Mirdamadi and Block, 2020). Given the absence of changes in SAI in this study, albeit without direct evidence evaluating sensorimotor connectivity, whole-hand WF did not alter sensorimotor connectivity and integration, potentially leading to no significant impact on motor memory retention. Another conceivable explanation pertains to the plastic response elicited by the current preconditioning. In a study targeting patients with stroke, Celnik et al. (2007) reported that LTP-like plasticity induced by RSS facilitated motor memory retention, accompanied by a reduction in intracortical inhibition. Conversely, previous studies that documented the facilitation of motor memory retention through plastic changes in the S1 and M1 among healthy participants employed protocols for LTD-like plasticity responses, as opposed to LTP-like, revealing that disruptions in the S1 or M1 compromised memory retention (Richardson et al., 2006; Darainy et al., 2023). This indicates that in individuals with normal plasticity, a saturation effect may hinder the observation of facilitative effects of LTP-like induction protocols.

There are several limitations to the interpretation of the results in this study. Firstly, the use of a pinching movement in the motor task, where the FDI was not the primary muscle, may have confounded the interpretation. Future studies should explore this issue using motor tasks involving index finger abduction. Secondly, the repetition of the same task during motor learning may have led to a ceiling effect on performance, potentially limiting the generalizability of the findings. Future research with more difficult tasks is needed. Thirdly, investigating the effects of whole-hand WF with varying preconditioning durations could have provided valuable insights. Although the duration of 30 min for preconditioning was chosen based on prior findings, its impact on skill acquisition and retention during sensorimotor adaptation remained unclear. Thus, exploring different preconditioning durations may have yielded different results. Finally, this study only examined the effects of a single preconditioning session. Several studies have found that multiple sessions of perceptual learning enhance experience-dependent plasticity (Trzcinski et al., 2016); however, the effects of multiple sessions of RSS alone on sensorimotor adaptation remain uncertain. Therefore, investigating the effects of multiple sessions would be intriguing. In conclusion, the present results suggested that whole-hand WF did not modulate skill acquisition or motor memory retention during sensorimotor adaptation; however, this preconditioning attenuated intracortical inhibition.

## Data availability statement

The raw data supporting the conclusions of this article will be made available by the authors, without undue reservation.

## Ethics statement

The studies involving humans were approved by Ethics Committee of Niigata University of Health and Welfare. The studies were conducted in accordance with the local legislation and institutional requirements. The participants provided their written informed consent to participate in this study.

## Author contributions

DL: Data curation, Formal analysis, Investigation, Writing – original draft, Writing – review & editing. DS: Conceptualization, Data curation, Formal analysis, Funding acquisition, Investigation, Methodology, Project administration, Resources, Software, Supervision, Validation, Visualization, Writing – original draft, Writing – review & editing. KI: Formal analysis, Investigation, Methodology, Project administration, Resources, Software, Writing – original draft, Writing – review & editing. GO: Investigation, Methodology, Writing – review & editing. TF: Investigation, Methodology, Writing – review & editing. KY: Conceptualization, Formal analysis, Investigation, Methodology, Resources, Supervision, Writing – original draft, Writing – review & editing.

## References

[ref1] Baraduc P. Lang N. Rothwell J. C. Wolpert D. M. (2004). Consolidation of dynamic motor learning is not disrupted by rTMS of primary motor cortex. Curr. Biol. 14, 252–256. doi: 10.1016/j.cub.2004.01.033, PMID: 14761660

[ref2] Barr D. J. Levy R. Scheepers C. Tily H. J. (2013). Random effects structure for confirmatory hypothesis testing: keep it maximal. J. Mem. Lang. 68, 255–278. doi: 10.1016/j.jml.2012.11.001, PMID: 24403724 PMC3881361

[ref3] Bernardi N. F. Darainy M. Ostry D. J. (2015). Somatosensory contribution to the initial stages of human motor learning. J. Neurosci. 35, 14316–14326. doi: 10.1523/JNEUROSCI.1344-15.2015, PMID: 26490869 PMC4683690

[ref4] Bütefisch C. M. Davis B. C. Wise S. P. Sawaki L. Kopylev L. Classen J.. (2000). Mechanisms of use-dependent plasticity in the human motor cortex. Proc. Natl. Acad. Sci. USA 97, 3661–3665. doi: 10.1073/pnas.97.7.3661 10716702 PMC16296

[ref5] Cantarero G. Lloyd A. Celnik P. (2013). Reversal of long-term potentiation-like plasticity processes after motor learning disrupts skill retention. J. Neurosci. 33, 12862–12869. doi: 10.1523/JNEUROSCI.1399-13.2013, PMID: 23904621 PMC3728692

[ref6] Celnik P. Hummel F. Harris-Love M. Wolk R. Cohen L. G. (2007). Somatosensory stimulation enhances the effects of training functional hand tasks in patients with chronic stroke. Arch. Phys. Med. Rehabil. 88, 1369–1376. doi: 10.1016/j.apmr.2007.08.001, PMID: 17964875

[ref7] Classen J. Steinfelder B. Liepert J. Stefan K. Celnik P. Cohen L. G.. (2000). Cutaneomotor integration in humans is somatotopically organized at various levels of the nervous system and is task dependent. Exp. Brain Res. 130, 48–59. doi: 10.1007/s002210050005, PMID: 10638440

[ref8] Darainy M. Manning T. F. Ostry D. J. (2023). Disruption of somatosensory cortex impairs motor learning and retention. J. Neurophysiol. 130, 1521–1528. doi: 10.1152/jn.00231.2023, PMID: 37964765

[ref9] Darainy M. Vahdat S. Ostry D. J. (2013). Perceptual learning in sensorimotor adaptation. J. Neurophysiol. 110, 2152–2162. doi: 10.1152/jn.00439.2013, PMID: 23966671 PMC4073967

[ref10] Della-Maggiore V. Villalta J. I. Kovacevic N. McIntosh A. R. (2017). Functional evidence for memory stabilization in sensorimotor adaptation: a 24-h resting-state fMRI study. Cereb. Cortex 27, 1748–1757. doi: 10.1093/cercor/bhv289, PMID: 26656723

[ref11] Doyon J. Benali H. (2005). Reorganization and plasticity in the adult brain during learning of motor skills. Curr. Opin. Neurobiol. 15, 161–167. doi: 10.1016/j.conb.2005.03.004 15831397

[ref12] Fothergill D. M. Taylor W. F. Hyde D. E. (1998). Physiologic and perceptual responses to hypercarbia during warm- and cold-water immersion. Undersea Hyperb. Med. 25, 1–12, PMID: 9566081

[ref13] Gedankien T. Fried P. J. Pascual-Leone A. Shafi M. M. (2017). Intermittent theta-burst stimulation induces correlated changes in cortical and corticospinal excitability in healthy older subjects. Clin. Neurophysiol. 128, 2419–2427. doi: 10.1016/j.clinph.2017.08.034, PMID: 29096215 PMC5955003

[ref14] Gentilucci M. Toni I. Daprati E. Gangitano M. (1997). Tactile input of the hand and the control of reaching to grasp movements. Exp. Brain Res. 114, 130–137. doi: 10.1007/PL00005612, PMID: 9125458

[ref15] Godde B. Spengler F. Dinse H. R. (1996). Associative pairing of tactile stimulation induces somatosensory cortical reorganization in rats and humans. Neuroreport 8, 281–285. doi: 10.1097/00001756-199612200-00056, PMID: 9051796

[ref16] Golaszewski S. M. Siedentopf C. M. Baldauf E. Koppelstaetter F. Eisner W. Unterrainer J.. (2002). Functional magnetic resonance imaging of the human sensorimotor cortex using a novel vibrotactile stimulator. NeuroImage 17, 421–430. doi: 10.1006/nimg.2002.1195, PMID: 12482095

[ref17] Hadipour-Niktarash A. Lee C. K. Desmond J. E. Shadmehr R. (2007). Impairment of retention but not acquisition of a visuomotor skill through time-dependent disruption of primary motor cortex. J. Neurosci. 27, 13413–13419. doi: 10.1523/JNEUROSCI.2570-07.2007, PMID: 18057199 PMC6673085

[ref18] Hamel R. Trempe M. Bernier P.-M. (2017). Disruption of M1 activity during performance plateau impairs consolidation of motor memories. J. Neurosci. 37, 9197–9206. doi: 10.1523/JNEUROSCI.3916-16.2017, PMID: 28821677 PMC6596746

[ref19] Jones E. G. (1983). The nature of the afferent pathways conveying short-latency inputs to primate motor cortex. Adv. Neurol. 39, 263–285, PMID: 6318531

[ref20] Kaelin-Lang A. Luft A. R. Sawaki L. Burstein A. H. Sohn Y. H. Cohen L. G. (2002). Modulation of human corticomotor excitability by somatosensory input. J. Physiol. 540, 623–633. doi: 10.1113/jphysiol.2001.012801, PMID: 11956348 PMC2290238

[ref21] Kakigi R. (1994). Diffuse noxious inhibitory control. Reappraisal by pain-related somatosensory evoked potentials following CO2 laser stimulation. J. Neurol. Sci. 125, 198–205. doi: 10.1016/0022-510X(94)90036-1, PMID: 7807168

[ref22] Krakauer J. W. Hadjiosif A. M. Xu J. Wong A. L. Haith A. M. (2019). Motor learning. Compr. Physiol. 9, 613–663. doi: 10.1002/cphy.c170043, PMID: 30873583

[ref23] Kreidler S. M. Muller K. E. Grunwald G. K. Ringham B. M. Coker-Dukowitz Z. T. Sakhadeo U. R.. (2013). GLIMMPSE: online power computation for linear models with and without a baseline covariate. J. Stat. Softw. 54:i10. doi: 10.18637/jss.v054.i10, PMID: 24403868 PMC3882200

[ref24] Kujirai T. Caramia M. D. Rothwell J. C. Day B. L. Thompson P. D. Ferbert A.. (1993). Corticocortical inhibition in human motor cortex. J. Physiol. 471, 501–519. doi: 10.1113/jphysiol.1993.sp019912, PMID: 8120818 PMC1143973

[ref25] Kumar N. Manning T. F. Ostry D. J. (2019). Somatosensory cortex participates in the consolidation of human motor memory. PLoS Biol. 17:e3000469. doi: 10.1371/journal.pbio.3000469, PMID: 31613874 PMC6793938

[ref26] Le Cong D. Sato D. Ikarashi K. Fujimoto T. Ochi G. Yamashiro K. (2022). Effect of whole-hand water flow stimulation on the neural balance between excitation and inhibition in the primary somatosensory cortex. Front. Hum. Neurosci. 16:962936. doi: 10.3389/fnhum.2022.962936, PMID: 36393986 PMC9640458

[ref27] Mirdamadi J. L. Block H. J. (2020). Somatosensory changes associated with motor skill learning. J. Neurophysiol. 123, 1052–1062. doi: 10.1152/jn.00497.2019, PMID: 31995429

[ref28] Muellbacher W. Ziemann U. Wissel J. Dang N. Kofler M. Facchini S.. (2002). Early consolidation in human primary motor cortex. Nature 415, 640–644. doi: 10.1038/nature712 11807497

[ref29] Nakatani-Enomoto S. Hanajima R. Hamada M. Terao Y. Matsumoto H. Shirota Y.. (2012). Bidirectional modulation of sensory cortical excitability by quadripulse transcranial magnetic stimulation (QPS) in humans. Clin. Neurophysiol. 123, 1415–1421. doi: 10.1016/j.clinph.2011.11.037, PMID: 22280937

[ref30] Négyesi J. Veldman M. P. Berghuis K. M. M. Javet M. Tihanyi J. Hortobágyi T. (2018). Somatosensory electrical stimulation does not augment motor skill acquisition and Intermanual transfer in healthy young adults-a pilot study. Mot. Control. 22, 67–81. doi: 10.1123/mc.2016-0048, PMID: 28338389

[ref31] Ohashi H. Gribble P. L. Ostry D. J. (2019). Somatosensory cortical excitability changes precede those in motor cortex during human motor learning. J. Neurophysiol. 122, 1397–1405. doi: 10.1152/jn.00383.2019, PMID: 31390294 PMC6843109

[ref32] Paparella G. Rocchi L. Bologna M. Berardelli A. Rothwell J. (2020). Differential effects of motor skill acquisition on the primary motor and sensory cortices in healthy humans. J. Physiol. 598, 4031–4045. doi: 10.1113/JP279966 32639599

[ref33] Pfenninger C. Zeghoudi N. Bertrand M. F. Lapole T. (2024). Effects of prolonged vibration to the flexor carpi radialis muscle on intracortical excitability. Sci. Rep. 14:8475. doi: 10.1038/s41598-024-59255-5, PMID: 38605084 PMC11009410

[ref34] Reuter E.-M. Booms A. Leow L.-A. (2022). Using EEG to study sensorimotor adaptation. Neurosci. Biobehav. Rev. 134:104520. doi: 10.1016/j.neubiorev.2021.104520 35016897

[ref35] Richardson A. G. Overduin S. A. Valero-Cabré A. Padoa-Schioppa C. Pascual-Leone A. Bizzi E.. (2006). Disruption of primary motor cortex before learning impairs memory of movement dynamics. J. Neurosci. 26, 12466–12470. doi: 10.1523/JNEUROSCI.1139-06.2006, PMID: 17135408 PMC6674906

[ref36] Ridding M. C. McKay D. R. Thompson P. D. Miles T. S. (2001). Changes in corticomotor representations induced by prolonged peripheral nerve stimulation in humans. Clin. Neurophysiol. 112, 1461–1469. doi: 10.1016/S1388-2457(01)00592-2, PMID: 11459686

[ref37] Ridding M. C. Taylor J. L. Rothwell J. C. (1995). The effect of voluntary contraction on cortico-cortical inhibition in human motor cortex. J. Physiol. 487, 541–548. doi: 10.1113/jphysiol.1995.sp020898, PMID: 8558482 PMC1156591

[ref38] Rocchi L. Erro R. Antelmi E. Berardelli A. Tinazzi M. Liguori R.. (2017). High frequency somatosensory stimulation increases sensori-motor inhibition and leads to perceptual improvement in healthy subjects. Clin. Neurophysiol. 128, 1015–1025. doi: 10.1016/j.clinph.2017.03.046, PMID: 28463818

[ref39] Rosenkranz K. Rothwell J. C. (2012). Modulation of proprioceptive integration in the motor cortex shapes human motor learning. J. Neurosci. 32, 9000–9006. doi: 10.1523/JNEUROSCI.0120-12.2012, PMID: 22745499 PMC6622352

[ref40] Rossi S. Hallett M. Rossini P. M. Pascual-Leone A. Safety of TMS Consensus Group (2009). Safety, ethical considerations, and application guidelines for the use of transcranial magnetic stimulation in clinical practice and research. Clin. Neurophysiol. 120, 2008–2039. doi: 10.1016/j.clinph.2009.08.016 19833552 PMC3260536

[ref41] Rusu C. V. Murakami M. Ziemann U. Triesch J. (2014). A model of TMS-induced I-waves in motor cortex. Brain Stimul. 7, 401–414. doi: 10.1016/j.brs.2014.02.009, PMID: 24680789

[ref42] Sato D. Yamashiro K. Onishi H. Baba Y. Nakazawa S. Shimoyama Y.. (2014a). Whole-body water flow stimulation to the lower limbs modulates excitability of primary motor cortical regions innervating the hands: a transcranial magnetic stimulation study. PLoS One 9:e102472. doi: 10.1371/journal.pone.0102472, PMID: 25025129 PMC4099321

[ref43] Sato D. Yamashiro K. Onishi H. Yasuhiro B. Shimoyama Y. Maruyama A. (2014b). Whole-hand water flow stimulation increases motor cortical excitability: a study of transcranial magnetic stimulation and movement-related cortical potentials. J. Neurophysiol. 113, 822–833. doi: 10.1152/jn.00161.2014 25376780

[ref44] Sato D. Yamashiro K. Yoshida T. Onishi H. Shimoyama Y. Maruyama A. (2013). Effects of water immersion on short- and long-latency afferent inhibition, short-interval intracortical inhibition, and intracortical facilitation. Clin. Neurophysiol. 124, 1846–1852. doi: 10.1016/j.clinph.2013.04.008, PMID: 23688919

[ref45] Tokimura H. Di Lazzaro V. Tokimura Y. Oliviero A. Profice P. Insola A.. (2000). Short latency inhibition of human hand motor cortex by somatosensory input from the hand. J. Physiol. 523, 503–513. doi: 10.1111/j.1469-7793.2000.t01-1-00503.x, PMID: 10699092 PMC2269813

[ref46] Trzcinski N. K. Gomez-Ramirez M. Hsiao S. S. (2016). Functional consequences of experience-dependent plasticity on tactile perception following perceptual learning. Eur. J. Neurosci. 44, 2375–2386. doi: 10.1111/ejn.13343, PMID: 27422224 PMC5028271

[ref47] Turco C. V. Toepp S. L. Foglia S. D. Dans P. W. Nelson A. J. (2021). Association of short- and long-latency afferent inhibition with human behavior. Clin. Neurophysiol. 132, 1462–1480. doi: 10.1016/j.clinph.2021.02.402, PMID: 34030051

[ref48] Veldman M. P. Maffiuletti N. A. Hallett M. Zijdewind I. Hortobágyi T. (2014). Direct and crossed effects of somatosensory stimulation on neuronal excitability and motor performance in humans. Neurosci. Biobehav. Rev. 47, 22–35. doi: 10.1016/j.neubiorev.2014.07.013, PMID: 25064816

[ref49] Veldman M. P. Maurits N. M. Zijdewind I. Maffiuletti N. A. van Middelkoop S. Mizelle J. C.. (2018). Somatosensory electrical stimulation improves skill acquisition, consolidation, and transfer by increasing sensorimotor activity and connectivity. J. Neurophysiol. 120, 281–290. doi: 10.1152/jn.00860.2017, PMID: 29641307

[ref50] Veldman M. P. Zijdewind I. Maffiuletti N. A. Hortobágyi T. (2016). Motor skill acquisition and retention after somatosensory electrical stimulation in healthy humans. Front. Hum. Neurosci. 10:115. doi: 10.3389/fnhum.2016.00115 27014043 PMC4792880

[ref51] Wu C. W.-H. van Gelderen P. Hanakawa T. Yaseen Z. Cohen L. G. (2005). Enduring representational plasticity after somatosensory stimulation. NeuroImage 27, 872–884. doi: 10.1016/j.neuroimage.2005.05.055, PMID: 16084740

[ref52] Yildiz F. G. Temucin C. M. (2023). Multimodal integration and modulation of visual and somatosensory inputs on the corticospinal excitability. Neurophysiol. Clin. 53:102842. doi: 10.1016/j.neucli.2022.102842, PMID: 36724583

[ref53] Ziemann U. Tergau F. Wassermann E. M. Wischer S. Hildebrandt J. Paulus W. (1998). Demonstration of facilitatory I wave interaction in the human motor cortex by paired transcranial magnetic stimulation. J. Physiol. 511, 181–190. doi: 10.1111/j.1469-7793.1998.181bi.x, PMID: 9679173 PMC2231091

